# Molecular Classification Based on Prognostic and Cell Cycle-Associated Genes in Patients With Colon Cancer

**DOI:** 10.3389/fonc.2021.636591

**Published:** 2021-04-07

**Authors:** Zhiyuan Zhang, Meiling Ji, Jie Li, Qi Wu, Yuanjian Huang, Guodong He, Jianmin Xu

**Affiliations:** ^1^Department of General Surgery, Zhongshan Hospital, Fudan University, Shanghai, China; ^2^Department of General Surgery, The First Affiliated Hospital of Nanjing Medical University, Nanjing, China

**Keywords:** colon cancer, cell cycle, non-negative matrix factorization, unsupervised cluster, molecular subtypes

## Abstract

The molecular classification of patients with colon cancer is inconclusive. The gene set enrichment analysis (GSEA) of dysregulated genes among normal and tumor tissues indicated that the cell cycle played a crucial role in colon cancer. We performed univariate Cox regression analysis to find out the prognostic-related genes, and these genes were then intersected with cell cycle-associated genes and were further recognized as prognostic and cell cycle-associated genes. Unsupervised non-negative matrix factorization (NMF) clustering was performed based on cell cycle-associated genes. Two subgroups were identified with different overall survival, clinical features, cell cycle enrichment profile, and mutation profile. Through nearest template prediction (NTP), the molecular classification could be effectively repeated in the original data set and validated in several independent data sets indicating that the classification is highly repeatable. Furthermore, we constructed two prognostic signatures in two subgroups, respectively. Our molecular classification based on cell cycle may provide novel insight into the treatment and the prognosis of colon cancer.

## Introduction

Colon cancer is widely recognized as one of the most common cancers worldwide. Due to the rapid progress in the treatment and diagnosis of colon cancer, mortality has decreased significantly in recent years. However, the potential mechanism of colon cancer has not been clarified till now. In addition, in the era of precision treatment for certain cancers, according to molecular subtypes classified by molecular characteristics, patients receive different treatments and show different prognostic outcomes. For example, patients with breast cancer are often classified into three subtypes according to the molecular characteristics, namely the expression of estrogen or progesterone receptors and human epidermal growth factor 2 (ERBB2, also named HER2). Different patients show diverse risk profiles, and the therapy strategy will differ according to the subtype. Overall, the molecular classification provides great value in the treatment and assessment of the prognosis of patients with breast cancer (1). In colon cancer, the consensus molecular subtypes (CMS) are relatively commonly recognized (2). However, the classification of colon cancer in the molecular layer does not show a similar clinical value to breast cancer, indicating that CMS still has some deficiency in the clinical translation. Thus, it is quite urgent to identify the potential molecular subtypes to aid the treatment and prognostic assessment of patients with colon cancer.

In the present research, we first identified the differentially expressed genes (DEGs) between normal [normal colon samples from the Genotype–Tissue Expression (GTEx) data sets and tumor-adjacent normal colon samples from the cancer genome atlas (TCGA)] and tumor samples [colon cancer samples in The Cancer Genome Atlas (TCGA)]. The gene set enrichment analysis (GSEA) indicated that the dysregulated genes were mainly enriched with cell cycle-associated gene sets (Hallmark E2F targets, Hallmark MYC targets, and Hallmark G2M checkpoint). We supposed that the cell cycle might play a significant role in colon cancer.

The cell cycle is one of the most important biological processes in the human body (3). The cell cycle mainly involves regulating the cell division and duplication of genetic materials. It is also highly associated with the proliferation of cells. For the most commonly recognized views, the cell cycle contains four continuous phases, which were characterized as G0/G1, S, G2, and M phases (4). The G1/G0 phase is the process before DNA replication, and it is also recognized as the pre-synthesis period. This period mainly functions to synthesize the RNA and ribosomes. In the S phase, DNA replication is more frequent; besides, histones and enzymes, which are fundamental for DNA replication, are also created in this phase. G2 is the preparatory phase for mitosis and is recognized as the end of DNA synthesis. The M phase involves the phase of mitosis in which the cell often divides into two daughter cells. The M phase also contains four sequential periods, including prophase, metaphase, anaphase, and telophase. The transition of these four phases is tightly regulated by the interaction between several cyclin-dependent kinases (CDKs) and their corresponding cyclin partners (5). The cell cycle is also an indispensable component that participates almost in the entire process of cancer. An important characteristic of cancer is uncontrolled cell proliferation, which is mainly regulated by the cell cycle. Dysregulated cell cycle regulators were reported to play important roles in diverse cancers (6, 7). In colon cancer, previous studies also demonstrate that the cell cycle plays a vital function in the initiation and progress of cancer (8–10). Because of the significant role of the cell cycle in cancers, we hypothesize that colon cancer could be clustered as different molecular subtypes based on cell cycle-associated genes.

In the present study, we identified two molecular subtypes according to the cell cycle-related genes. These two molecular groups showed different genetic mutations, overall survival (OS), and clinical features. Moreover, through nearest template prediction (NTP), the molecular classification could be effectively repeated in the original data set and validated in several independent data sets indicating that the classification is highly repeatable. Our molecular classification based on the cell cycle may provide novel insight into the treatment and prognosis of colon cancer.

## Materials and Methods

### Process on the Original Data

Raw microarray information of colon cancer with matched clinical information and normal colon samples were obtained from TCGA database, Gene Expression Omnibus (GEO), and GTEx database. The data from TCGA and GTEx were obtained from Xena (http://xena.ucsc.edu/), and in order to conduct the representational difference analysis (RDA), the data from GTEx and TCGA were normalized according to the description of individual data sets from Xena and then combined together. In GEO data sets, the selection criteria were set as below: (1) data sets should be built through the Affymetrix HG-U133 plus 2.0 platform; (2) data sets should at least have clinical information about the American Joint Committee on Cancer (AJCC) staging system, OS interval, and OS status; and (3) larger samples in the individual data set are preferred where there are least 200 samples or more. Finally, GSE17538 and GSE39582 complied with the criterion (11, 12).

### Representational Difference Analysis and GSEA

Dysregulated genes between normal and tumor tissues were determined through the Wilcoxon test based on R language (Version 3.6.2). In this study, the normal tissues consisted of normal colon tissues from the GTEx database, colon cancer tissues and adjacent normal tissues were obtained from the TCGA database, and the tumor tissues were obtained from the colon cancer samples from the TCGA database. GSEA was conducted through R language (Version 3.6.2) and “h: the hallmark gene sets,” that were downloaded from MSigDB (https://www.gsea-msigdb.org/gsea/index.jsp), was selected as the gene reference set.

### Identification of the Prognostic and Cell Cycle-Associated Genes

The cell cycle-associated genes were downloaded from MSigDB (https://www.gsea-msigdb.org/gsea/index.jsp). Genes involved in “KEGGCELLCYCLE,” which were filtered from “KEGG gene sets as Gene Symbols” in “c2: curated gene sets,” were selected, and genes involved in “GOCELLCYCLE,” which were also picked from “all GO gene sets as Gene Symbols” in “c5: Ontology gene sets,” were also picked. The genes of these two reference gene sets were merged and deduplicated and identified as cell cycle-associated genes. According to the clinical information from TCGA, univariate Cox regression analysis was performed on the micro RNAs (mRNAs), genes with outlier hazard ratio (HR) values are deleted, and genes with *p* < 0.05 and HR < 0.8 or HR > 1.2 were screened out and were intersected with cell cycle-associated genes; the results were defined as prognostic and cell cycle-associated genes. The process was carried out through the R language (Version 3.6.2).

### Identification and Validation About the Molecular Subgroups

Based on the prognostic and cell cycle-associated genes, the unsupervised non-negative matrix factorization (NMF) clustering method was carried out on the TCGA database through the R package, “Cancer Subtypes” (13). The optimal number of clusters (k) was comprehensively considered according to the molecular clustering effects and cophenetic correlation analysis. The survival analysis was conducted through a Kaplan–Meier plot and log-rank test. For individual molecular subgroups, RDA was conducted to identify the top 50 upregulated hall markers between other molecular subgroups through the R package, “LIMMA.” NTP was then carried out based on the top 50 upregulated markers of the individual molecular subgroup. The NTP was first conducted in the TCGA database to show the accuracy of the prediction based on the NTP method. Then, the NTP was carried out in GSE17538 and GSE39582 data sets. The clinical features, including gender, T, N, M, and AJCC stages, and survival profile in the individual molecular subgroup, were compared among diverse data sets to assess the repeatability and accuracy of the molecular subgroup prediction based on NTP.

### Degree of Enrichment of Cell Cycle in Diverse Molecular Subgroups

The cell cycle-associated gene sets, which were statistically significant in the previous GSEA analysis, were included and identified as reference gene sets for single sample gene set enrichment analysis (ssGSEA) to analyze the degree of enrichment of cell cycle among the diverse molecular subgroups. Each sample was computed according to the ssGSEA analysis through the R language (Version 3.6.2). The RDA among diverse molecular subgroups was analyzed by the R package “LIMMA,”

### Mutation Profile Between Different Molecular Subgroups

The mutation data of patients with chronic obstructive airway disease (COAD) was obtained from the TCGA data set (https://portal.gdc.cancer.gov/). Maftools were utilized to analyze the data. The mutation data were visualized and analyzed based on the two different molecular subgroups.

### Construction of Two Overall-Survival Prognostic Signatures in Two Subgroups, Respectively

In individual subgroups, the patients included in the GEO data sets were randomly divided into two groups with a ratio of 7:3. About 70% of patients were identified as belonging to an inter-training group, and 30% of patients were identified as belonging to an inter-test group. Prognostic and cell cycle-associated genes were processed through univariate Cox regression analysis in two subgroups, respectively. The genes that owned HR > 1.2 or HR < 0.8 and *p* < 0.05 were involved in the least absolute shrinkage and selection operator (LASSO) analysis. Then the respective signatures were constructed. The signatures were validated in a test group based on TCGA data sets. The area under the curve (AUC) of the receiver operating characteristic curve (ROC) was used to evaluate the efficiency of these signatures.

## Results

### Dysregulated Genes Among Normal and Tumor Samples and Function Enrichment Analysis

**Figure 1:**
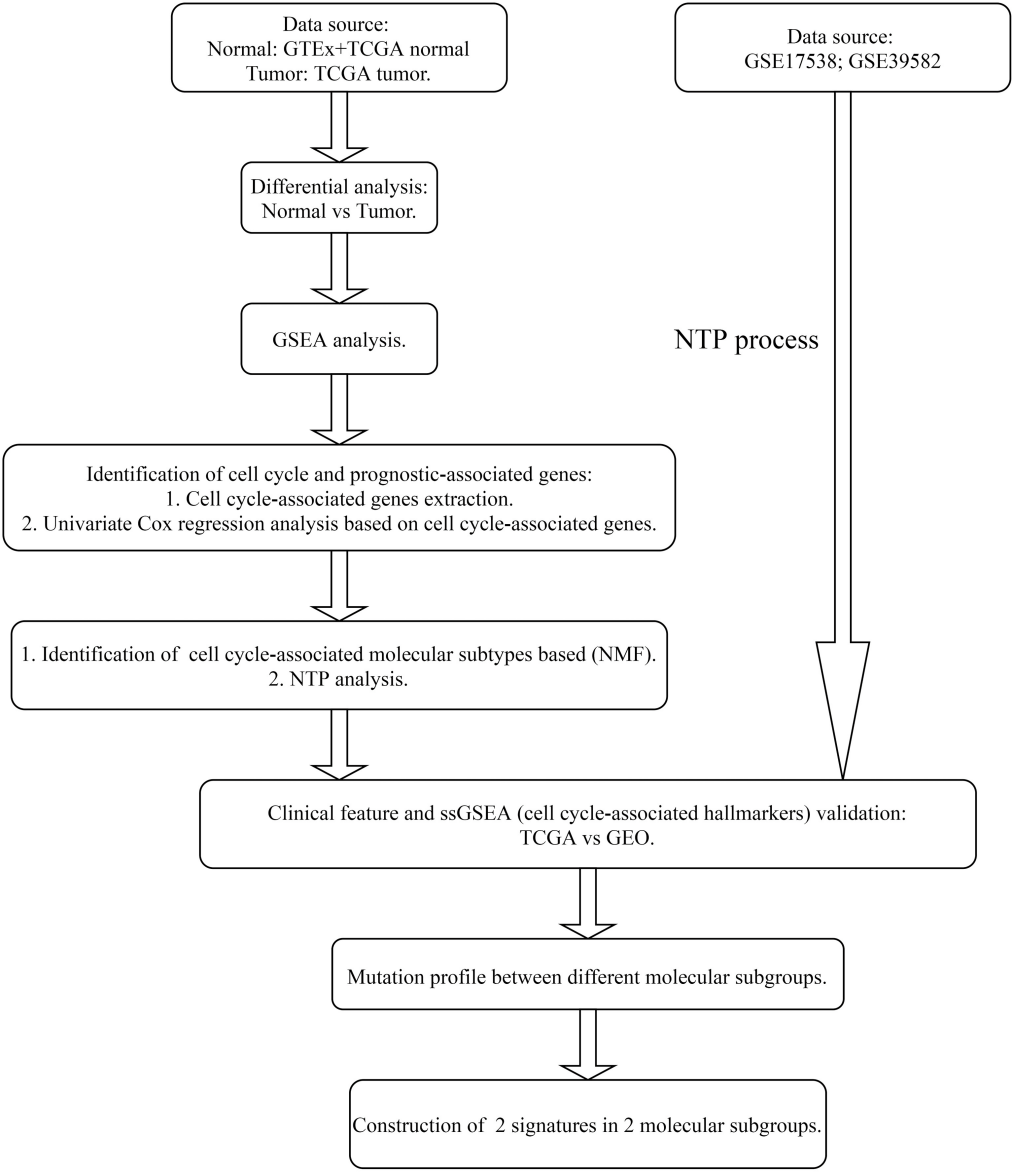
Analysis flow of this research.

**Figure 2:**
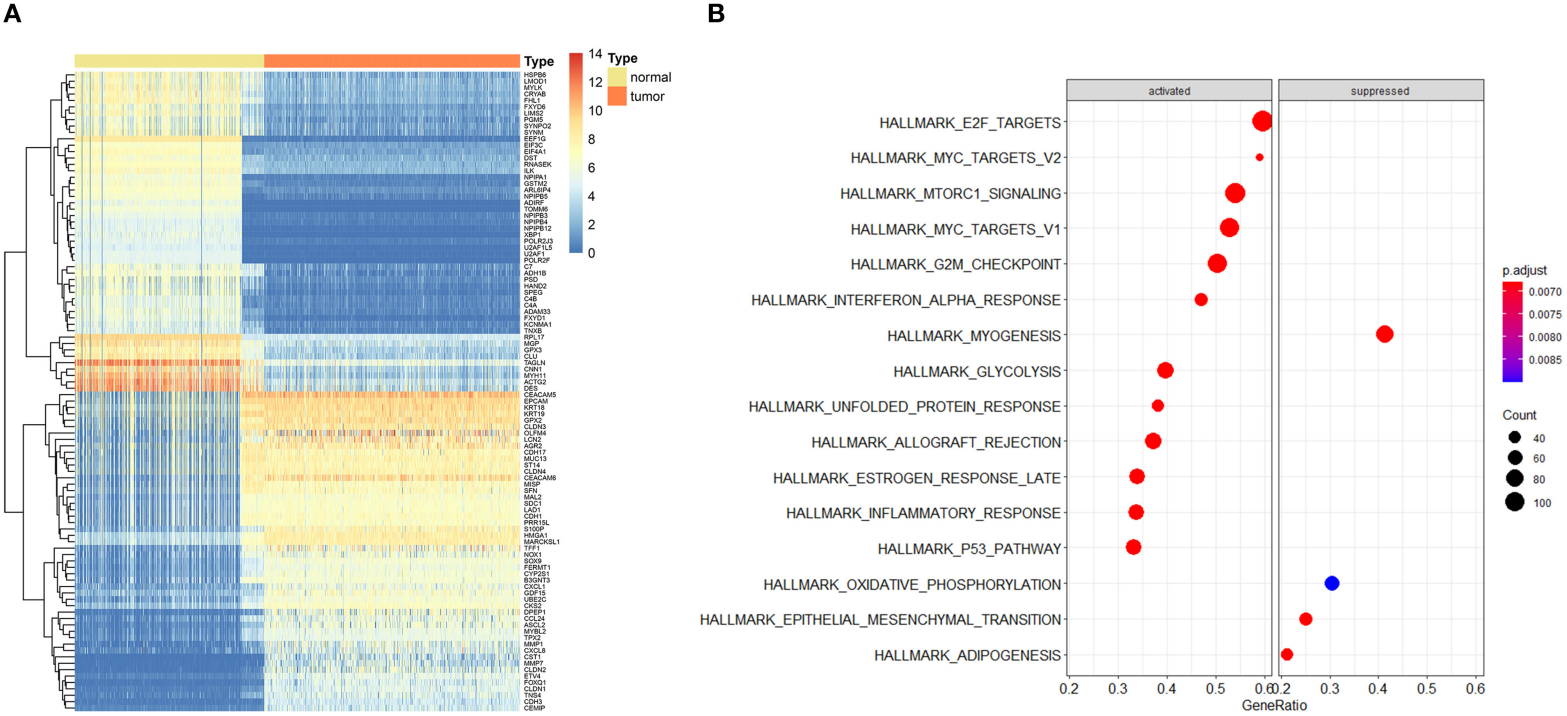
Expression profile of top dysregulated genes between normal and tumor samples and gene set enrichment analysis (GSEA). **(A)** Heatmap depicted the expression profile of the top 50 upregulated and top 50 downregulated genes between normal and tumor tissues. **(B)** GSEA results of differentially expressed genes.

The entire analysis flow is presented in Figure 1. We integrally analyzed the dysregulated genes among normal and tumor tissues. In the current study, the normal tissues consisting of normal colon tissues were obtained from the GTEx database, colon cancer-adjacent normal tissues were obtained from the TCGA database, and the tumor tissues were obtained from colon cancer samples from the TCGA database. We have represented the expression profile of the top 50 upregulated and downregulated genes between normal and cancer tissues in the form of a heatmap (Figure 2A). After identifying the DEGs, we then performed GSEA to research the functional enrichment of these DEGs. The results indicated that several cell cycle-associated gene sets were mainly enriched and activated by E2F targets, MYC targets, G2M checkpoint, and the P53 pathway, and the four gene sets were suppressed including myogenesis, oxidative phosphorylation, epithelial–mesenchymal transition, and adipogenesis. Other gene sets that were high ranked include mTORC1 signaling, interferon–alpha response, and glycolysis (Figure 2B).

### Identification of the Molecular Subgroups

**Figure 3:**
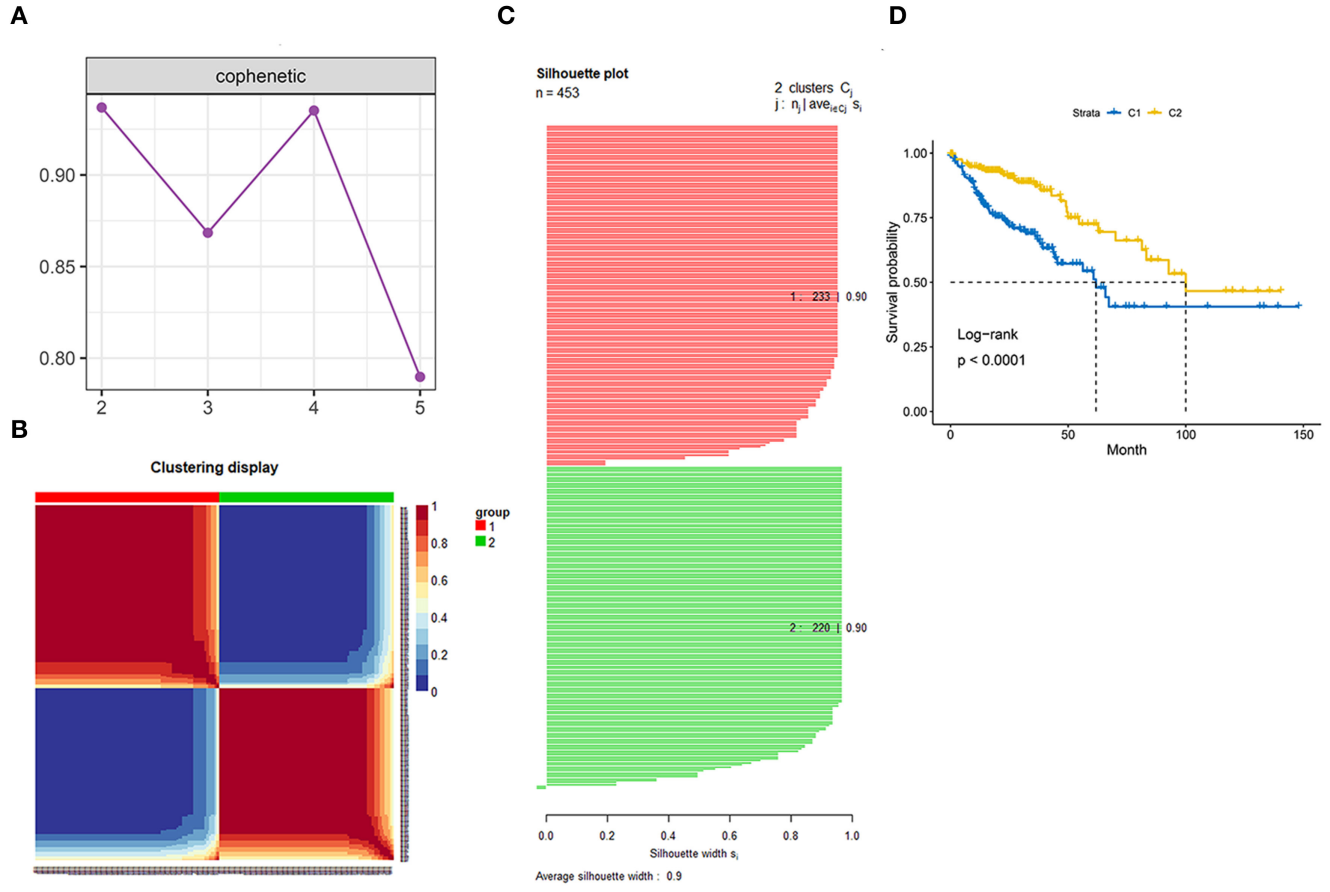
Identification of the molecular subgroups. **(A)** Cophenetic correlation analysis. **(B)** Clustering display of the molecular subgroups. **(C)** Silhouette plot of the molecular subgroups. **(D)** Overall survival (OS) of the two molecular subgroups.

Genes involved in the gene sets named, “KEGG gene sets as Gene Symbols” and “GO_CELL_CYCLE” were combined and determined as cell cycle-associated genes. Finally, 1,875 genes were recognized as cell cycle-associated genes (Supplementary Table 1). Then, based on the OS information from the TCGA, a univariate Cox regression analysis was performed on all the micro RNAs (mRNAs). The filter criterion was set as HR <0.8 or HR >1.2 with *p* < 0.05, genes with outlier HR were excluded (Supplementary Table 2), and the results were intersected with cell cycle-associated genes. Finally, 149 genes were determined as cell cycle and prognostic-associated genes (Supplementary Table 3). Unsupervised NMF clustering was conducted based on the 149 genes. The optimal k was comprehensively considered according to the molecular clustering effects and cophenetic correlation analysis. We tried k from 2 to5, and the results of clustering, OS analysis, and silhouette plots when *k* = 3–5 are presented in Supplementary Figures 1–3. When *k* = 2, the clustering results, OS analysis, and silhouette plots presented better effects (Figures 3A–D). Moreover, cophenetic correlation analysis showed that *k* = 3 dropped a lot compared to when *k* = 2. Hence, *k* = 2 was preferred, and the two subgroups were identified as C1 and C2, respectively.

### Validation of the Molecular Subgroup Classified by NTP

**Figure 4:**
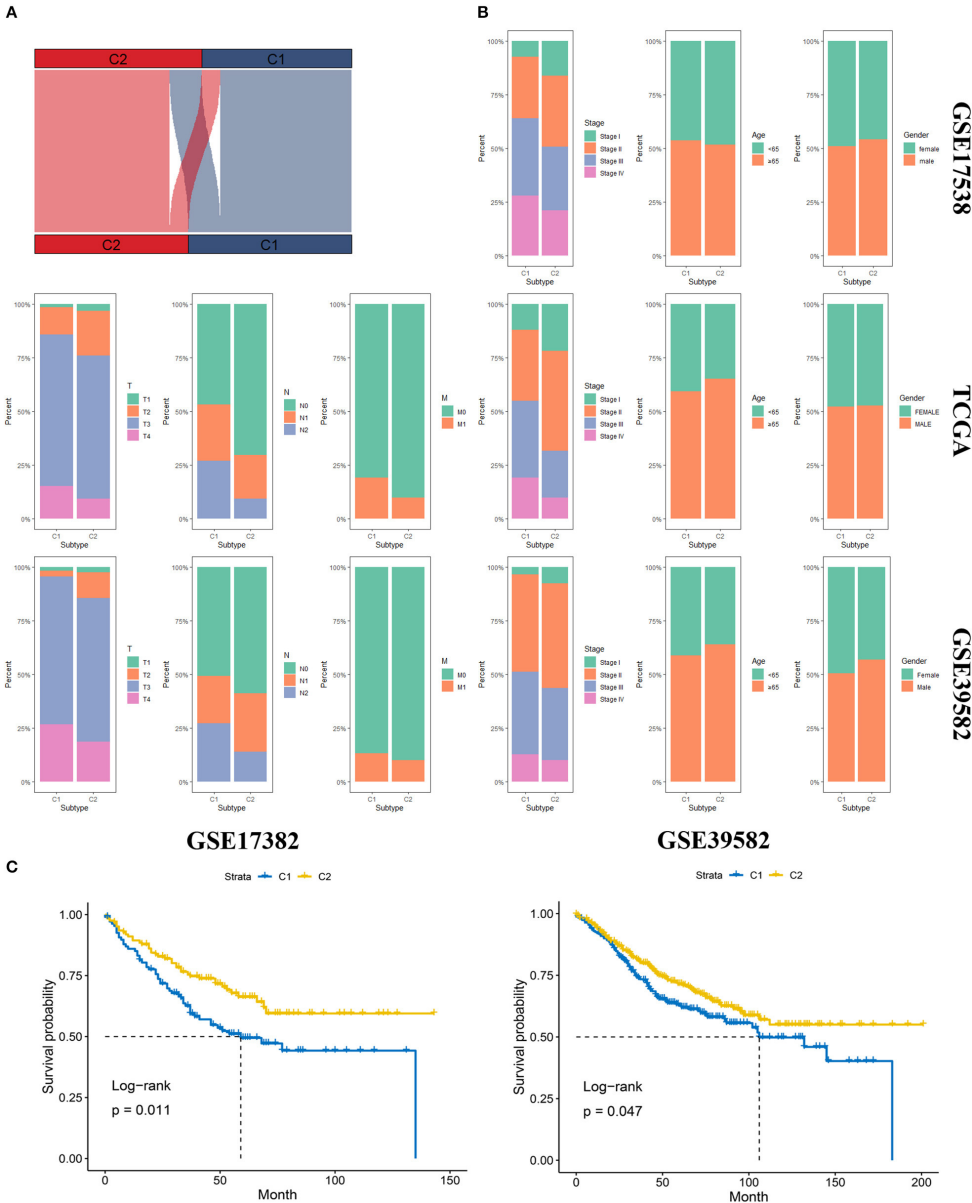
Validation of the molecular subgroup classified by nearest template prediction (NTP). **(A)** Sankey plot depicted the accuracy of the NTP method. Upper layer represents the original subgroups, and the down layer represents the subgroups predicted by NTP. **(B)** The distribution of several clinical features, including AJCC stage, age, gender, T stage, N status, and M status in GSE17538, TCGA, and GSE39582. **(C)** The Overall survival analysis of the two molecular subgroups in diverse data sets.

We first identified the top 50 upregulated genes for an individual group compared to the other group. These top 50 upregulated genes were recognized as markers of the corresponding subgroups (Supplementary Table 4). Then, NTP analysis was first conducted on the TCGA database, and the result of the clustering was compared to the original clustering and the comparison showed a high accuracy of NTP for the prediction of subgroups (Figure 4A). Later, we performed NTP on GSE17538 and GSE39582, and distribution of several clinical features included AJCC stage, age, gender, T stage, N status, and M status (Figure 4B). The OS was also compared between the two molecular subgroups in diverse data sets (Figure 4C). The distribution and survival analysis indicated that the molecular subgroups showed similar clinical features among different data sets *in* terms of age and gender, and the two molecular subgroups were similar; however, in terms of T stage, N and M status, and AJCC stage, C1 showed later stage compared to C2. Correspondingly, C1 showed worse OS compared to C2. The process showed that the molecular subgroups could be repeated based on the NTP method with high accuracy. In addition, the subgroups predicted by NTP were highly consistent with the previous subgroups that were classified based on the TCGA database.

## Results of ssGSEA Between Two Subgroups in Terms of Cell Cycle

**Figure 5:**
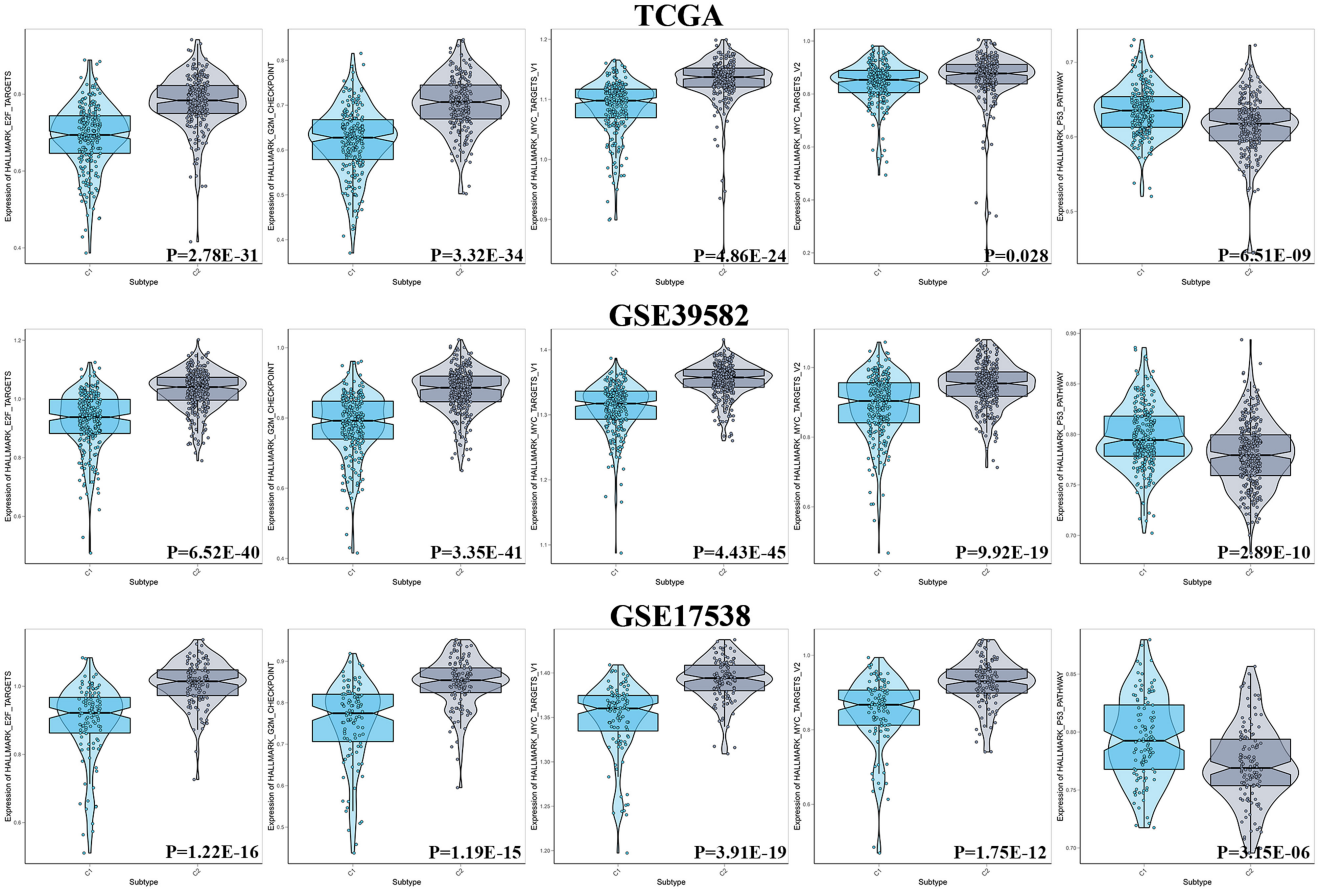
Results of ssGSEA between two subgroups in terms of cell cycle.

The cell cycle-associated gene sets that were statistically significant in the previous GSEA analysis were included and identified as reference gene sets for ssGSEA to analyze the degree of enrichment of cell cycle among the diverse molecular subgroups. The results showed that no matter in TCGA, GSE17538, or GSE39582, E2F targets, G2M checkpoint, and MYC targets were more enriched with C2, and in terms of the P53 pathway, C1 was more enriched (Figure 5).

### Mutation Profile Between Different Molecular Subgroups

**Figure 6:**
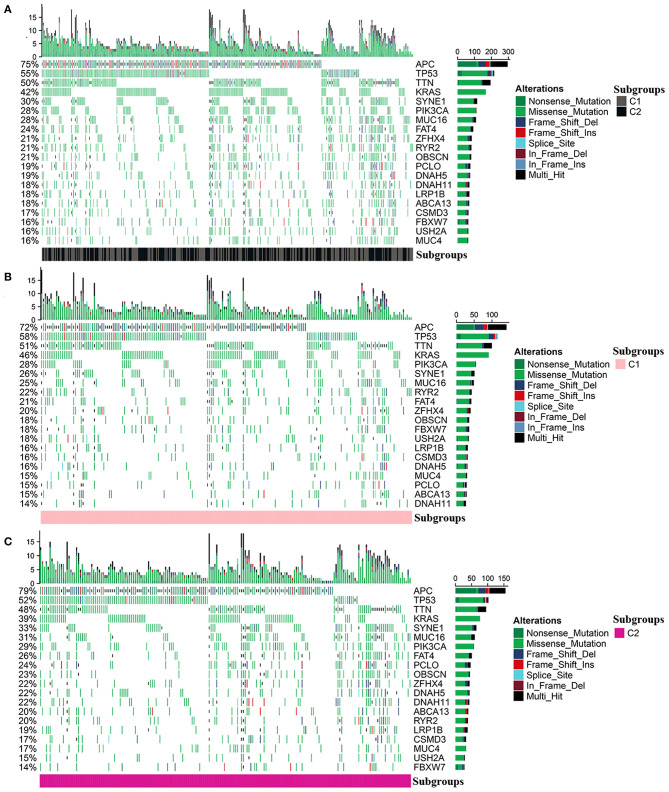
Mutation profile in the entire colon cancer and two subgroups. **(A)** The mutation profile in all patients with colon cancer. **(B,C)** The mutation profile in C1 and C2.

The mutation profile was integrated and then analyzed according to the diverse subgroups, respectively (Figure 6A). The APC, TP53, TTN, KRAS, SYNE1, and PIK3CA were the top five frequently mutated genes in colon cancer. But the mutations differed between C1 and C2. In C1 and C2, the top four frequently mutated genes remained as APC, TP53, TTN, and KRAS; however, mutations in PIK3CA, RYR2, and FBXW7 were more frequent in C1 compared to C2. SYNE1, PCLO, and MUC16 were more frequently mutated in C2 (Figures 6B,C).

## Construction of Two OS Prognostic Signatures in Two Subgroups, Respectively

**Figure 7:**
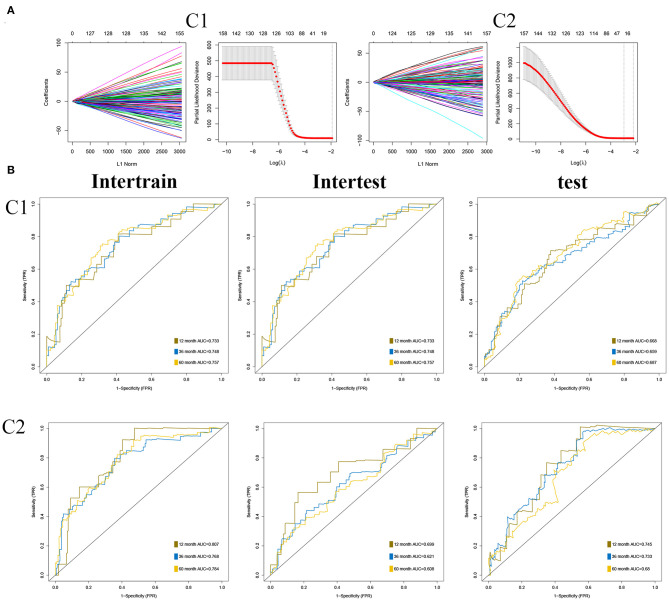
Construction of prognostic signatures in two subgroups, respectively. **(A)** Results of least absolute shrinkage and selection operator (LASSO) analysis in two subgroups. **(B)** Area under the curve (AUC) of receiver operating characteristic curve (ROC) of 2 signatures in two subgroups.

As described in Methods and Materials, we constructed two prognostic signatures in two subgroups, respectively. The results of the LASSO analysis are presented in Figure 7A. AUC was used to evaluate the efficiency of signatures in two subgroups (Figure 7B). In C1, the signature was presented as (expression of ASRGL1) ^*^ (−0.334) + (expression of CCL22) ^*^ (−0.578) + (expression of ERCC3) ^*^ 0.711 + (expression of F2RL2) ^*^ (−0.263) + (expression of HNF1B) ^*^ (−0.409) + (expression of MYB) ^*^ (−0.456) + (expression of PTTG1IP) ^*^ 0.365 + (expression of RIPK4) ^*^ 0.635. The signature in C2 was presented as (expression of ARMT1) ^*^ 0.752 + (expression of CCL22) ^*^ (−1.017) + (expression of CUZD1) ^*^ 0.626 + (expression of EPHB2) ^*^ (−0.552) + (expression of ERFE) ^*^ 0.353 + (expression of LYPD6) ^*^ (−0.785) + (expression of MS4A2) ^*^ (−1.626).

## Discussion

Colorectal cancer (CRC) ranks third in the estimation of morbidity and mortality among all cancer types as per the 2020 cancer statistics (14). Colon cancer is a common type of CRC. Due to the progress in colon cancer therapy and diagnosis, mortality has decreased significantly recently. However, the treatment for colon cancer is still less specific, and there is still much room for improvement for the therapy and diagnosis of colon cancer, as the molecular subtype for colon cancer is not clarified. In the era of precision medicine, the treatment for individual patients is specific and many patients who are diagnosed with some other types of cancer can benefit greatly due to the specific treatment (1). Colon cancer also has several molecular subtyping such as CMS (2), but it is based on patients with type I–III colon cancer, and it is still not wildly used in the clinical treatment, indicating that it has some deficiency in the clinical transition. According to the current status of the treatment of colon cancer, it is necessary to research the potential molecular subtyping in colon cancer to help improve the precise treatment for patients with colon cancer.

Benefiting from the advancement in bioinformatics, especially in the high-throughput sequencing technology, we can research the potential mechanism of cancers at the transcriptome level. Many researchers used bioinformatics tools to construct efficient signatures to help better estimate the prognosis and tried to reveal the latent mechanism of diverse cancers (15–21). Moreover, cluster analysis can classify patients with cancer into diverse molecular subtypes according to the expression pattern of specific gene sets that are identified as gene references. However, the kinds of gene sets that were set as references will be the decisive factor for the efficiency of molecular subtyping.

In the present study, based on the data from GTEx and TCGA, we used RDA to screen out the dysregulated genes between normal colon tissues and tissues from colon cancer. Through GSEA, we found that the cell cycle might have an important role in the process of colon cancer (the gene sets were enriched with E2F targets, MYC targets, the G2M checkpoint, and the P53 pathway); thus, the prognostic and cell cycle-associated genes were identified as references for molecular subtyping.

The cell cycle is a significant part of the biological process (22–25). Usually, cells proliferate only in response to developmental or other mitotic signals that are essential for normal tissue growth; however, the proliferation of cancer cells is not under control. This indicates that cancer cells proliferate due to the dysregulation of internal and external inhibition of proliferation signals that can be regulated by the cell cycle. Many previous studies proved that some dysregulated genes could mediate the expression of several key factors that could regulate the cell cycle of cancer cells. In conclusion, the cell cycle plays a crucial role in the occurrence and progress of the tumor and may also play a vital role in the molecular classification of patients with tumors. We hypothesized that cell cycle-associated genes could have great performance in molecular subgrouping and conducted the sequenced bioinformatic analysis.

Through repeated tests on the number of the optimal number of k, we finally identified two molecular subtypes that were named “C1” and “C2”; C1 has a worse prognosis in terms of OS than C2, and patients with C1 showed worse clinical characteristics, especially in the T stage, N status, M status, and AJCC stage, than C2. Moreover, the mutation profile differed in the two subtypes. Mutations in PIK3CA, RYR2, and FBXW7 were more frequent in C1 compared to C2. SYNE1, PCLO, and MUC16 were more frequently mutated in C2. The difference between the two groups of mutations might also differ in the treatment methods and drugs. For better clinical applications, we identified 50 upregulated genes as markers for individual subtypes. The NTP method was utilized to predict the molecular subtypes based on the gene markers, and this method showed great accuracy in prediction compared to the original molecular subtyping. In the independent data sets, such as GSE17538 and GSE39582, the subtypes predicated by NTP method also showed similar clinical features and clinical outcomes as the TCGA data sets; moreover, the enrichment profile of cell cycle for two subtypes in GSE17538, GSE39582, and TCGA also showed high similarity indicating that the molecular subtype based on prognostic and cell cycle-associated gene sets and the NTP method was efficient, accurate, and highly repeatable.

In conclusion, we used prognostic and cell cycle-associated genes to cluster patients with colon cancer into two types, where the two types of patients with colon cancer showed different mutation profile, clinical features, and OS outcomes. Moreover, we used the NTP method to highly and accurately repeat our molecular subtyping. The molecular subtypes based on prognostic and cell cycle-associated genes might give us insight into the treatment and the estimation of the prognosis of patients with colon cancer.

## Data Availability Statement

The original contributions generated for the study are included in the article/Supplementary Material, further inquiries can be directed to the corresponding authors.

## Author Contributions

ZZ, JX, and GH designed and conducted the study. ZZ and YH wrote the article. MJ and QW helped to improve and design the study. JL helped to improve the research. All authors contributed to the article and approved the submitted version.

## Conflict of Interest

The authors declare that the research was conducted in the absence of any commercial or financial relationships that could be construed as a potential conflict of interest.

## Supplementary Material

Supplementary Table 1 Cell cycle-related genes.

Supplementary Table 2 Micro RNAs (mRNAs) screened out according to univariate Cox regression analysis, the mRNAs with outlier HR were excluded and the filter was set as HR <0.8 or HR >1.2 and *p* < 0.05.

Supplementary Table 3 Prognostic and cell cycle-related Micro RNAs (mRNAs).

Supplementary Table 4 50 upregulated genes identified as markers of the corresponding subgroups.

Supplementary Figure 1 The results of overall survival (OS) analysis, clustering display, and silhouette plots when *k* = 3.

Supplementary Figure 2 The results of overall survival (OS) analysis, clustering display, and silhouette plots when *k* = 4.

Supplementary Figure 3 The results of overall survival (OS) analysis, clustering display, and silhouette plots when *k* = 5.
