# Supplementary figures and images for: Molecular Classification Based on Prognostic and Cell Cycle-Associated Genes in Patients With Colon Cancer
Source: Front Oncol. 2021 Apr 7;11:636591. doi: 10.3389/fonc.2021.636591 (PMC8059408; doi:10.3389/fonc.2021.636591)

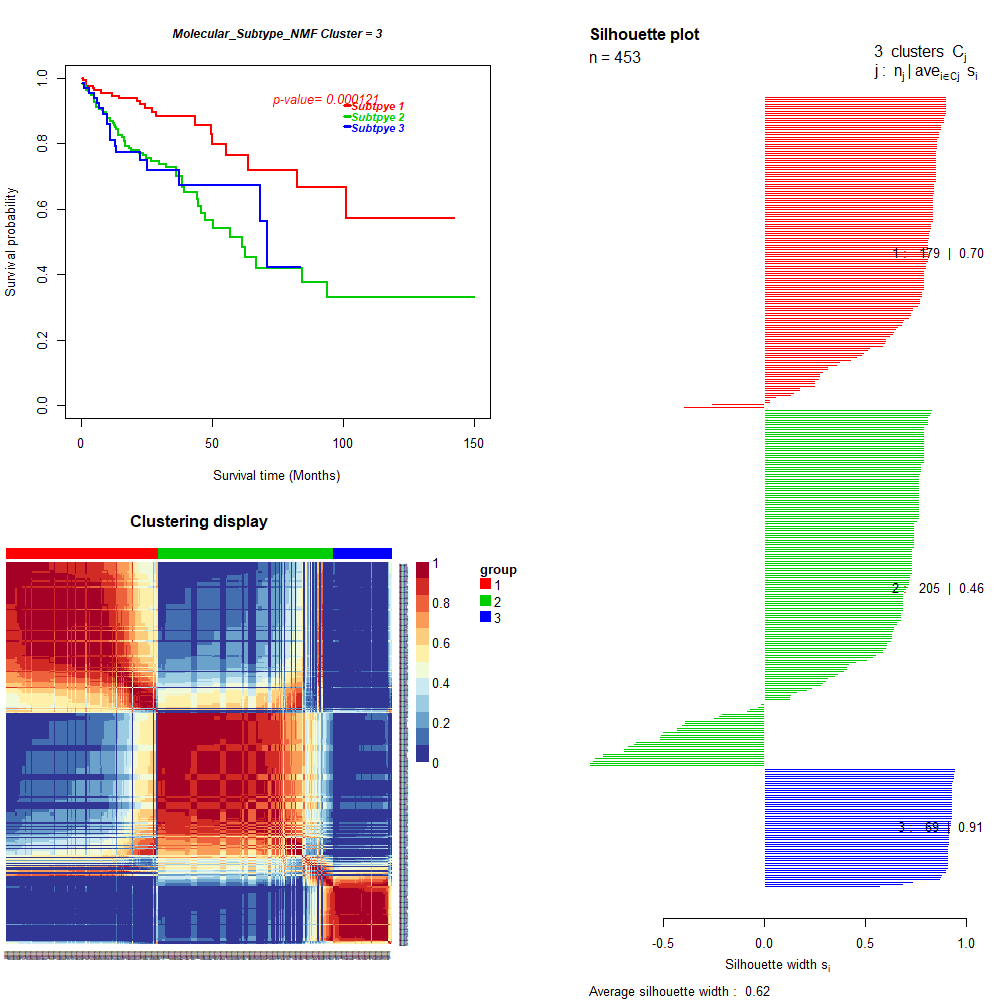

Supplement: Supplementary Figure 1 — The results of overall survival (OS) analysis, clustering display, and silhouette plots when k = 3. [file Image_1.TIFF]

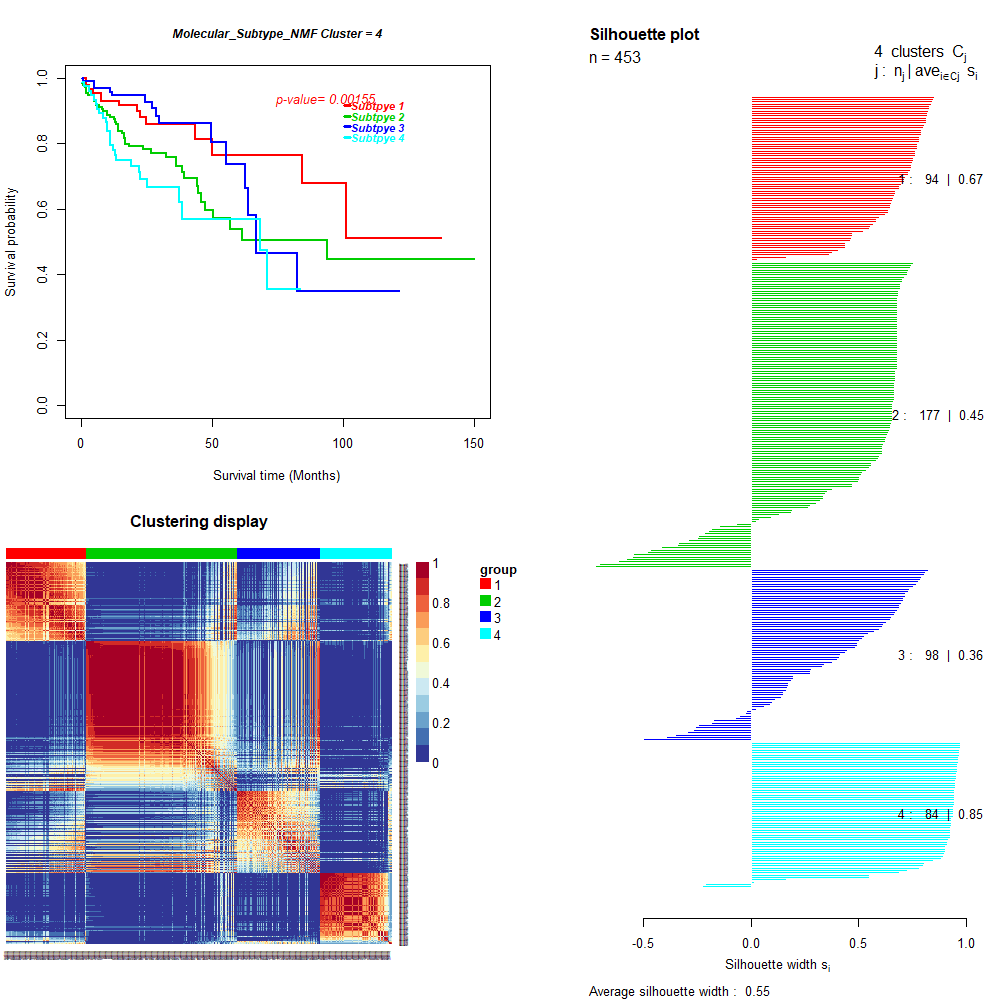

Supplement: Supplementary Figure 2 — The results of overall survival (OS) analysis, clustering display, and silhouette plots when k = 4. [file Image_2.TIFF]

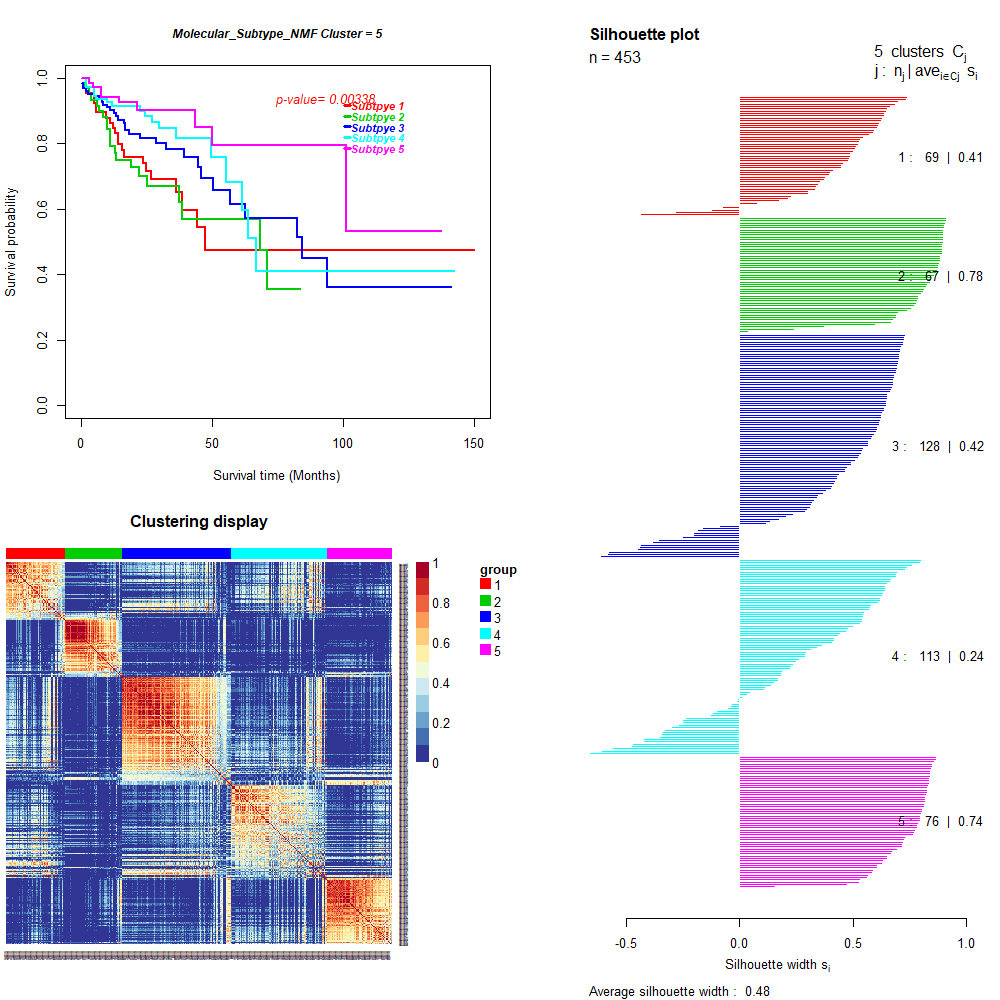

Supplement: Supplementary Figure 3 — The results of overall survival (OS) analysis, clustering display, and silhouette plots when k = 5. [file Image_3.TIFF]
